# Water Deficit Modulates the CO_2_ Fertilization Effect on Plant Gas Exchange and Leaf-Level Water Use Efficiency: A Meta-Analysis

**DOI:** 10.3389/fpls.2021.775477

**Published:** 2021-11-29

**Authors:** Fei Li, Dagang Guo, Xiaodong Gao, Xining Zhao

**Affiliations:** ^1^College of Water Resources and Architectural Engineering, Northwest A&F University, Xianyang, China; ^2^Key Laboratory of Agricultural Soil and Water Engineering in Arid and Semiarid Areas, Ministry of Education, Northwest A&F University, Xianyang, China; ^3^Institute of Soil and Water Conservation, Chinese Academy of Sciences and Ministry of Water Resources, Yangling, China; ^4^National Engineering Research Center of Water Saving and Irrigation Technology, Yangling, China; ^5^Institute of Soil and Water Conservation, Northwest A&F University, Xianyang, China

**Keywords:** CO_2_ fertilization effect, water deficit, leaf gas exchange, meta-analysis, graphical vector analysis

## Abstract

Elevated atmospheric CO_2_ concentrations ([eCO_2_]) and soil water deficits significantly influence gas exchange in plant leaves, affecting the carbon-water cycle in terrestrial ecosystems. However, it remains unclear how the soil water deficit modulates the plant CO_2_ fertilization effect, especially for gas exchange and leaf-level water use efficiency (WUE). Here, we synthesized a comprehensive dataset including 554 observations from 54 individual studies and quantified the responses for leaf gas exchange induced by e[CO_2_] under water deficit. Moreover, we investigated the contribution of plant net photosynthesis rate (*P*_*n*_) and transpiration rates (*T*_r_) toward WUE in water deficit conditions and e[CO_2_] using graphical vector analysis (GVA). In summary, e[CO_2_] significantly increased *P*_n_ and WUE by 11.9 and 29.3% under well-watered conditions, respectively, whereas the interaction of water deficit and e[CO_2_] slightly decreased *P*_n_ by 8.3%. Plants grown under light in an open environment were stimulated to a greater degree compared with plants grown under a lamp in a closed environment. Meanwhile, water deficit reduced *P*_n_ by 40.5 and 37.8%, while increasing WUE by 24.5 and 21.5% under ambient CO_2_ concentration (a[CO_2_]) and e[CO_2_], respectively. The e[CO_2_]-induced stimulation of WUE was attributed to the common effect of *P*_n_ and *T*_r_, whereas a water deficit induced increase in WUE was linked to the decrease in *T*_r_. These results suggested that water deficit lowered the stimulation of e[CO_2_] induced in plants. Therefore, fumigation conditions that closely mimic field conditions and multi-factorial experiments such as water availability are needed to predict the response of plants to future climate change.

## Introduction

Global atmospheric carbon dioxide concentration ([CO_2_]) has accelerated at an unprecedented pace of about 2.4 μmol mol^–1^ per year during the last decade, and presently, it is 413 ppm (IPCC, 2019; NASA, 2020). [CO_2_] is projected to be between 421–946 ppm by 2,100 depending on continued emission scenarios. This increase in [CO_2_] might be accompanied by shifting precipitation patterns and increasing extreme precipitation events (Bencze et al., 2014; Zhang et al., 2021). It is predicted that plants may be negatively affected by drought stress and yet may benefit from elevated CO_2_ (e[CO_2_]), known as the “CO_2_ fertilization effect.” An increase *P*_n_ and WUE are necessary for improve carbon-water cycle and plant productivity in terrestrial ecosystems. Therefore, understanding how soil water deficit affects “CO_2_ fertilization effect” on plant is of great significance to projecting the potential risk of climate change on global bio-environment equilibrium.

Generally, e[CO_2_] and drought stimulate or inhibit plant growth by changing leaf gas exchange including net photosynthesis rate (*P*_n_), transpiration rate (*T*_r_), stomatal conductance (*G*_*s*_), and leaf-level water use efficiency (WUE), which result in a significant impact on the global cycling of carbon-water in terrestrial ecosystems (McLaughlin et al., 2007; Lawson and Blatt, 2014). Water is a key reactant required by plants for various photochemical processes including as an electron donor in photosynthesis. Stomatal closure is one of the first visible drought tolerance mechanisms employed by plants to reduce excessive water loss (Hessini et al., 2009; Li et al., 2017). Drought reduces *G*_*s*_ by triggering abscisic acid (ABA) production in plants (Paoletti and Grulke, 2005; Yamaguchi et al., 2019; Li S. et al., 2020). Meanwhile, *G*_*s*_ is regulated by guard-cell water potential (Assmann, 1999; Raschke et al., 2003). Drought stress negatively affects plant physiology, which usually results in reduced *P*_n_, as *P*_n_ is closely associated with *G*_*s*_ and mesophyll conductance leading to CO_2_ diffusion, especially in C_3_ plants (Luomala et al., 2005; Haworth et al., 2013; Xu et al., 2016). Short-term water stress results in stomatal defense and increased WUE, which is associated with delayed drought (Ameye et al., 2012). Drought stress results in stomatal and non-stomatal limitations; for instance, a reduction in *P*_n_ may occur as a result of conditions favoring ribulose 1,5-bisphosphate (RuBP) oxygenation rather than carboxylation, resulting in a reduction of chlorophyll content (Farquhar and Sharkey, 1982; Ameye et al., 2012; Drake et al., 2017; Birami et al., 2020).

Atmospheric [CO_2_] is another pivotal factor affecting various biochemical processes of photosynthesis (Xu, 2015). Plants consume more CO_2_ to stimulate the carboxylation efficiency of ribulose-1,5-bisphosphate carboxylase oxygenase (Rubisco) to increase plant growth for most C_3_ plants while competitively reducing photorespiration and dark respiration when increasing atmosphere [CO_2_] (Leakey et al., 2006, 2007; Reddy et al., 2010; Robredo et al., 2010; Birami et al., 2020). However, the boost extent of photosynthesis induced by e[CO_2_] may be related to the plant growth environment. There are serious potential limitations in using enclosure systems when conducting experiments to explore the effect of elevated [CO_2_] on plants (Ainsworth, 2008). However, a study showed that the more closely fumigation conditions mimicked field conditions, the smaller was the stimulation of yield by elevated [CO_2_] (Ainsworth and Long, 2005). Additionally, some plants may begin to develop an adverse response to enriched CO_2_ environments, when beyond certain CO_2_ concentration limits (Cotrufo et al., 1998; Long et al., 2006; Taub et al., 2008; Xu, 2015). Meanwhile, the response of plant gas exchange to e[CO_2_] may be limited by other abiotic factors, such as high-temperature stress, low N/P, and low water availability (Bajji et al., 2001; Hessini et al., 2009; Zhao et al., 2016; Gorthi et al., 2019; Jiang et al., 2020). For example, high temperature can affect photosynthesis by reducing the activities of key metabolic enzymes in plants (Luomala et al., 2005); N deficiency can lead to inadequate sink strength and thus limit plant growth (Hyvönen et al., 2007); Severe drought conditions counteract e[CO_2_] improvements in physiological performance and yield in soybeans (Casteel et al., 2008). Additionally, in contrast to C_3_ plants, *P*_n_ of C_4_ plants may not be influenced by e[CO_2_] because [CO_2_] is not rate-limiting to photosynthesis (Zhang et al., 2021). However, under e[CO_2_], C_4_ species may have certain advantages over C_3_ plants regarding high WUE, especially under drought conditions (Barton et al., 2012). Studies have shown that WUE of maize and sorghum improved owing to direct or indirect stimulation of photosynthesis at e[CO_2_] (Allen et al., 2011; Yan et al., 2016), and other groups have reported that enhanced WUE results due to reduction in *G*_*s*_ and transpiration (Morison, 1985; Wu and Wang, 2000). Stomatal control is regulated by water stress, resulting in the maintenance of high plant water status at e[CO_2_] (Ferris and Taylor, 1994; Polley et al., 1999; Torralbo et al., 2019). However, contradictory results were found in *Brassica napus* regarding minimizing water loss by following the e[CO_2_]-induced stomatal closure (Faralli et al., 2017). Unfortunately, despite several studies on the response of plants to e[CO_2_], previous analyses have focused on how e[CO_2_] alleviates the adverse effects of drought on plant growth and physiology. Consequently, the impact of water deficit conditions in regulating the effect of e[CO_2_] on plants is poorly understood, particularly on photosynthesis and WUE.

Rising [CO_2_] levels in this century are predicted to stimulate the growth of C_3_ species, counteracting the negative impacts of greater drought on plant growth and crop yield (Parry et al., 2004; Bencze et al., 2014). However, a few studies have shown that the stimulation of crop yield by e[CO_2_] diminished to zero as drought intensified (Gray et al., 2016; Zheng et al., 2020). Conflicting results stemming from differences in experimental design have hampered the potential to draw general conclusions. In addition, as with several control experiments, many experimental tests for CO_2_ response have been conducted in a limited environment such as enclosed spaces and weak light intensity, which limits the inferences that can be drawn from previously published literature. These limitations highlight the need for quantitative analysis of the available experimental data to better predict and implement adaptation policies related to future climate change scenarios and limited water availability for agricultural irrigation. Here, we used meta-analysis to address three key questions: (1) To what extent does water deficit modulate the response of plant gas exchange to e[CO_2_]? (2) How does e[CO_2_] and water deficit affect leaf-level WUE, and what is the nature of the interaction between e[CO_2_] and water deficit? (3) Do these responses differ by plant type, photosynthetic pathway, and growing conditions? Meta-analysis was used to test the effects of water availability on plant responses to e[CO_2_] by hypothesizing that: the CO_2_ fertilization effect on photosynthesis is reduced by water deficit (H1); water deficit × e[CO_2_] has no effect on WUE because water availability has a greater effect on plants than that of e[CO_2_] (H2); the more closely the fumigation conditions mimic field conditions, the stronger the stimulation of *P*_n_ by e[CO_2_] (H3). Hypothesis H1 was generally consistent with previous studies on the mitigation of stress by e[CO_2_], while H2 and H3 have no consensus. In this study, the synthetic power of meta-analysis was combined with graphical vector analysis (GVA) to investigate the contribution of plant *P*_n_ and *T*_r_ to WUE under water deficit and e[CO_2_].

## Materials and Methods

### Literature Search and Study Selection

We used the Web of Science^1^ and China National Knowledge databases (CNKI^2^) to search for peer-reviewed papers related to water deficit × CO_2_ interactions in plants. Various keyword combinations were used for the search, including (drought OR water deficit OR water supply) AND (CO_2_ enrichment OR doubled ambient CO_2_ OR rising CO_2_ OR CO_2_ rise) AND (gas exchange OR plant physiology). A total of 554 observations from 54 published papers were included in this meta-analysis (Appendix and Supplementary Material).

Only experiments on e[CO_2_] and water deficit were included, and the few available studies simultaneously testing a third variable, such as temperature or nutrient levels, were omitted. Factorial experiments included at least two water treatments in addition to two CO_2_ concentration treatments. The “well-watered,” “water deficit,” “ambient [CO_2_]”(a[CO_2_]), and “elevated [CO_2_]” (e[CO_2_]) treatments followed the definition of the authors of the original experiment (Wang et al., 2015). Meanwhile, the two most common methods of water control were chosen: dry days and field water capacity (FWC). All studies included plants growing in pots. We extracted response variables for leaf gas exchange, including *P*_n_, *G*_*s*_, *T*_r_, and instantaneous WUE, which was calculated from *P*_n_/*T*_r_ by authors. For those studies that included only *P*_n_ and *T*_r_ data but not WUE, we supplemented the WUE values with the equation: WUE = *P*_n_/*T*_r_. Data were taken from tables or digitized from figures using the software GetData Graph Digitizer, 2008 (ver. 2.22, Russian Federation).

The factorial experiments included four treatments: (i) ambient CO_2_ (a[CO_2_]) + well-watered (C_*a*_W_*w*_); (ii) e[CO_2_] + well-watered (C_*e*_W_*w*_); (iii) a[CO_2_] + water deficit (C_*a*_W_*d*_); and (iv) e[CO_2_] + water deficit (C_*e*_W_*d*_). In our dataset, a[CO_2_] treatments ranged from 350 to 450 ppm, while e[CO_2_] ranged from 500 to 1,200 ppm. The e[CO_2_] treatments were grouped into seven categories: 500–550, 551–600, 601–650, 651–700, 701–750, 751–800, and >800 ppm. In addition, CO_2_ exposure was either in a closed environment (growth chamber, greenhouse, and glasshouse), or an open or semi-open environment; free-air CO_2_ enrichment (FACE) and open top chamber (OTC). Water treatments were categorized into well-watered and water deficit, with the water-deficit treatment grouped into three categories for FWC: 65–55, 54–40, and <40%. Dry days were not grouped because of the small amount of data. Additionally, several explanatory variables in our meta-analysis may affect leaf gas exchange under water deficit and e[CO_2_], including plant type (grass, tree, legume, shrub, or crop), source of light (lamp or solar), and photosynthetic pathways (C_3_ or C_4_) as described in the Supplementary Tables 1, 2.

### Meta-Analytical Methods

We considered a[CO_2_] and well-watered treatments as the baseline, whereas e[CO_2_] and water deficit were the experimental treatments. The individual effect sizes for water and CO_2_ manipulation (*r*_*w*_ and *r*_*c*_, respectively) were calculated as follows:


(1) rWaCO2=X¯Ca⁢WdX¯Ca⁢Wwundera[CO]2treatment,

(2) rWeCO2=X¯Ce⁢WdX¯Ce⁢Wwundere[CO]2treatment,

(3) $$r_{\text{C}}^{{\text{W}}_{\text{w}}}=\frac{{\bar{\text{X}}}_{{\text{C}}_{\text{e}}\,{\text{W}}_{\text{w}}}}{{\bar{\text{X}}}_{{\text{C}}_{\text{a}}\,{\text{W}}_{\text{w}}}}\,\mathrm{under}\,\mathrm{well}-\mathrm{watered}\,\mathrm{treatment},$$

(4) $$r_{\text{C}}^{{\text{W}}_{\text{d}}}=\frac{{\bar{\text{X}}}_{{\text{C}}_{\text{e}}\,{\text{W}}_{\text{d}}}}{{\bar{\text{X}}}_{{\text{C}}_{\text{a}}\,{\text{W}}_{\text{d}}}}\,\mathrm{under}\,\mathrm{water}-\mathrm{deficit}\,\mathrm{treatment},$$

where $\bar{\text{X}}$, C_*e*_, C_*a*_, W_*w*_, and W_*d*_ represented the mean, e[CO_2_], a[CO_2_], well-watered, and water-deficit treatments, respectively (Jiang et al., 2020). The water deficit × CO_2_ interaction term was calculated from factorial experiments as described by Lajeunesse (2011). The water deficit and e[CO_2_] interaction effect size, or the effect of water deficit on the e[CO_2_] responses was calculated as follows:


(5) $$r=\frac{{\bar{\text{X}}}_{{\text{C}}_{\text{e}}\,{\text{W}}_{\text{d}}}}{{\bar{\text{X}}}_{{\text{C}}_{\text{a}}\,{\text{W}}_{\text{d}}}}\,/\,\frac{{\bar{\text{X}}}_{{\text{C}}_{\text{e}}\,{\text{W}}_{\text{w}}}}{{\bar{\text{X}}}_{{\text{C}}_{\text{a}}\,{\text{W}}_{\text{w}}}}$$

*r* was log-transformed to linearize this metric as follows:


(6) $$\text{Ln}\,\left(r\right)=\mathrm{Ln}\,\left(\frac{{\bar{\text{X}}}_{{\text{C}}_{\text{e}}\,{\text{W}}_{\text{d}}}}{{\bar{\text{X}}}_{{\text{C}}_{\text{a}}\,{\text{W}}_{\text{d}}}}\right)-\mathrm{Ln}\,\left(\frac{{\bar{\text{X}}}_{{\text{C}}_{\text{e}}\,{\text{W}}_{\text{w}}}}{{\bar{\text{X}}}_{{\text{C}}_{\text{a}}\,{\text{W}}_{\text{w}}}}\right)$$

Based on the additive property of variance (Curtis and Wang, 1998; Baig et al., 2015; Jiang et al., 2020), the variance of the water deficit by the CO_2_ interaction (*v*) response ratio was calculated as follows:


(7) $$v=\frac{{\text{SD}}_{{\text{C}}_{\text{a}}\,{\text{W}}_{\text{w}}}^{2}}{{\text{n}}_{{\text{C}}_{\text{a}}\,{\text{W}}_{\text{w}}}\,{\bar{\text{X}}}_{{\text{C}}_{\text{a}}\,{\text{W}}_{\text{w}}}^{2}}+\frac{{\text{SD}}_{{\text{C}}_{\text{e}}\,{\text{W}}_{\text{w}}}^{2}}{{\text{n}}_{{\text{C}}_{\text{e}}\,{\text{W}}_{\text{w}}}\,{\bar{\text{X}}}_{{\text{C}}_{\text{e}}\,{\text{W}}_{\text{w}}}^{2}}+\frac{{\text{SD}}_{{\text{C}}_{\text{a}}\,{\text{W}}_{\text{d}}}^{2}}{{\text{n}}_{{\text{C}}_{\text{a}}\,{\text{W}}_{\text{d}}}\,{\bar{\text{X}}}_{{\text{C}}_{\text{a}}\,{\text{W}}_{\text{d}}}^{2}}+\frac{{\text{SD}}_{{\text{C}}_{\text{e}}\,{\text{W}}_{\text{d}}}^{2}}{{\text{n}}_{{\text{C}}_{\text{e}}\,{\text{W}}_{\text{d}}}\,{\bar{\text{X}}}_{{\text{C}}_{\text{e}}\,{\text{W}}_{\text{d}}}^{2}}$$

An overall interaction term was estimated using weighted means, with greater weighting given to experiments with greater precision. A random-effects model was selected because the between-study variance was statistically significant. A multivariate linear mixed-effects model was then used to estimate the mean and the 95% confidence interval (CI) of the log-transformed response ratios for each individual variable, weighted by the variance of individual studies described in Supplementary Tables 1, 2. Effect sizes were reported as the antilog *r* converted to the mean percentage change from the baseline treatment [(*r* − 1) × 100]. Meta-analysis and Ln(*r*) calculations were conducted using OpenMEE software for ecological and evolutionary meta-analysis (Wallace et al., 2017).

### Graphic Vector Analysis Methodology

Graphical vector analysis was first developed to simultaneously compare the effect of experimental treatments on plant biomass, nutrient concentration, and nutrient content using an integrated graph (Haase and Rose, 1995). It is widely used because the comparison may be interpreted independently of predetermined critical levels or ratios.

In this analysis, GVA was used according to Wang et al. (2015); Couture et al. (2017), and Li Z. et al. (2020) to complement the meta-analysis of WUE. The magnitude of change in leaf-level WUE is determined by *P*_n_ and *T*_r_. Responses of leaf-level WUE to the experimental treatment relative to the control fell into one of the following seven categories:

(1) “Steady-state” increase, where WUE is unchanged owing to the parallel increases in leaf *P*_n_ and *T*_r_,
(2) “Steady-state” decrease, where WUE is unchanged owing to the parallel decreases in leaf *P*_n_ and *T*_r_,
(3) “*T*_r_” positive effect, where WUE increases because *T*_r_ decreases faster than *P*_n_,
(4) “*T*_r_” negative effect, where WUE decreases because *T*_r_ increases faster than *P*_n_,
(5) “*P*_n_” positive effect, where WUE increases because *P*_n_ increases faster than *T*_r_,
(6) “*P*_n_” negative effect, where WUE decreases because *P*_n_ decreases faster than *T*_r_,
(7) *P*_n_ and *T*_r_ work together, where WUE decreases/increases, despite *P*_n_ and *T*_r_ remaining unchanged, or *P*_n_ increases and *T*_r_ decreases.

All variables were plotted to illustrate the percent change in water deficit, e[CO_2_], and their interactions (value at C_*a*_W_*d*_ divided by those at C_*a*_W_*w*_ and multiplied by 100, value at C_*e*_W_*w*_ divided by those at C_*a*_W_*w*_ and multiplied by 100, value at C_*e*_W_*d*_ divided by those at C_*a*_W_*w*_ and multiplied by 100). *T*_r_ (*y*-axis) was plotted against *P*_n_ (*x*-axis) with WUE as the diagonal axis (*y*-axis) in a square-shaped diagram. Plotting the *P*_n_ data and WUE data automatically positioned the points along the diagonal lines representing the *z*-axis value for *T*_r_, allowing simultaneous examination of the three related variables on a two-dimensional diagram.

## Results

### Overview of Data Availability

**FIGURE 1:**
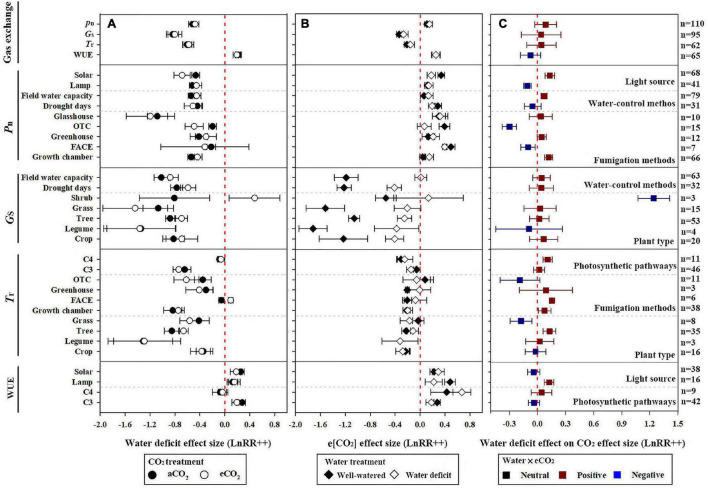
Effect of water deficit and elevated CO_2_ (e[CO_2_]) on plant gas exchange variables. **(A)** Effect of water deficit under ambient CO_2_ (a[CO_2_]), black circle, and e[CO_2_], white circle; **(B)** effect of e[CO_2_] under well-watered (white diamond) and water deficit (black diamond) conditions; and **(C)** effect of water deficit on plant response to e[CO_2_]: red, blue, and black boxes represent positive, negative, and statistically neutral effects, respectively. Response variables are as follows: net photosynthesis (*P*_n_), stomatal conductance (*G*_*s*_), transpiration rate (*T*_r_), and leaf-level water use efficiency (WUE = *P*_n_/*T*_r_). Water-deficit and e[CO_2_] treatment effect on plant *P*_n_ was classified according to light source, control methods of water, and CO_2_ fumigation methods; *G*_*s*_ was classified according to plant type and control methods of water; *T*_r_ was classified according to photosynthetic pathways, plant type, and CO_2_ fumigation methods; and WUE was classified according to light source and photosynthetic pathways (all separated by dotted lines). Dots and error bars represent means and 95% CI, respectively, of the log-transformed response ratio estimated based on a random effect model. LnRR(++) represent weighted mean response ratios. Number of data entries for each variable is denoted as *n*, labeled on the right *y*-axis. The treatment response was significant (*p* < 0.05) if the CI did not intersect with the red vertical dotted line on each plot (*x* = 0).

The dataset was derived from 54 studies of 554 valid data points of gas exchange (*P*_n_, *G*_*s*_, *T*_r_, and WUE) under e[CO_2_] and water deficit. Under e[CO_2_], *P*_n_ was increased by an average of 11.9 and 16.4% for well-watered and water-deficit treatments, respectively, for all plants, whereas the interaction of water deficit and e[CO_2_] slightly decreased *P*_n_ by 8.3%. That is, the e[CO_2_]-induced stimulation of *P*_n_ was reduced by the water deficit (Figures 1B,C). In contrast, water deficit reduced *P*_n_ by 40.5 and 37.8% because of a[CO_2_] and e[CO_2_] treatments, respectively, and WUE was increased by 24.5 and 21.5% (Figure 1B). The negative effects of water deficit on plant *P*_n_ were relieved at e[CO_2_] (Figures 1A–C).

In contrast to *P*_n_, e[CO_2_] reduced *G*_*s*_ and *T*_r_ by 23.3 and 14.4% under water-deficit treatments, respectively. However, a greater e[CO_2_] reduction was observed in well-watered plants (28.5 and 19.1%, respectively). Similarly, water-deficit treatment reduced *G*_*s*_ and *T*_r_ by 56.7 and 45.2%, respectively, under e[CO_2_] treatment (Figures 1A,B). In comparison, WUE was increased due to the e[CO_2_] and water deficit. e[CO_2_] increased WUE by 29.3 and 28.8% under well-watered and water-deficit treatments, respectively, and water deficit increased WUE by 24.5 and 21.5%, respectively, under a[CO_2_] and e[CO_2_] treatment (Figures 1A,B). In addition, the interaction between water deficit and e[CO_2_] and the impact on *G*_*s*_, *T*_r_, and WUE were not statistically significant.

### Net Photosynthetic Rate Responses

*P*_n_ responded to changes in water and CO_2_ treatments, types of light sources, and fumigation methods. The stimulation of *P*_n_ by e[CO_2_] was 50.7% lower under water-deficit treatments than the well-watered treatments using solar radiation (Figure 1), whereas the stimulation of CO_2_ on *P*_n_ under water deficit was almost the same as that under well-watered treatment using lamp radiation. Additionally, under e[CO_2_] conditions, water deficit decreased plant *P*_n_ by 41.8 and 35.6% when using the field capacity and drought days methods to control the water conditions, respectively. A greater increase in *P*_n_ was observed when plants were exposed to e[CO_2_] in a free-air CO_2_ enrichment system (FACE, 64.2%) than in closed glasshouses (35.3%), greenhouses (14.1%), or growth chambers (4.3%) (Figure 1B). However, the e[CO_2_]-induced increase in *P*_n_ was significantly reduced by water deficit compared with well-watered plants in any growing environment (Figure 1C).

**FIGURE 2:**
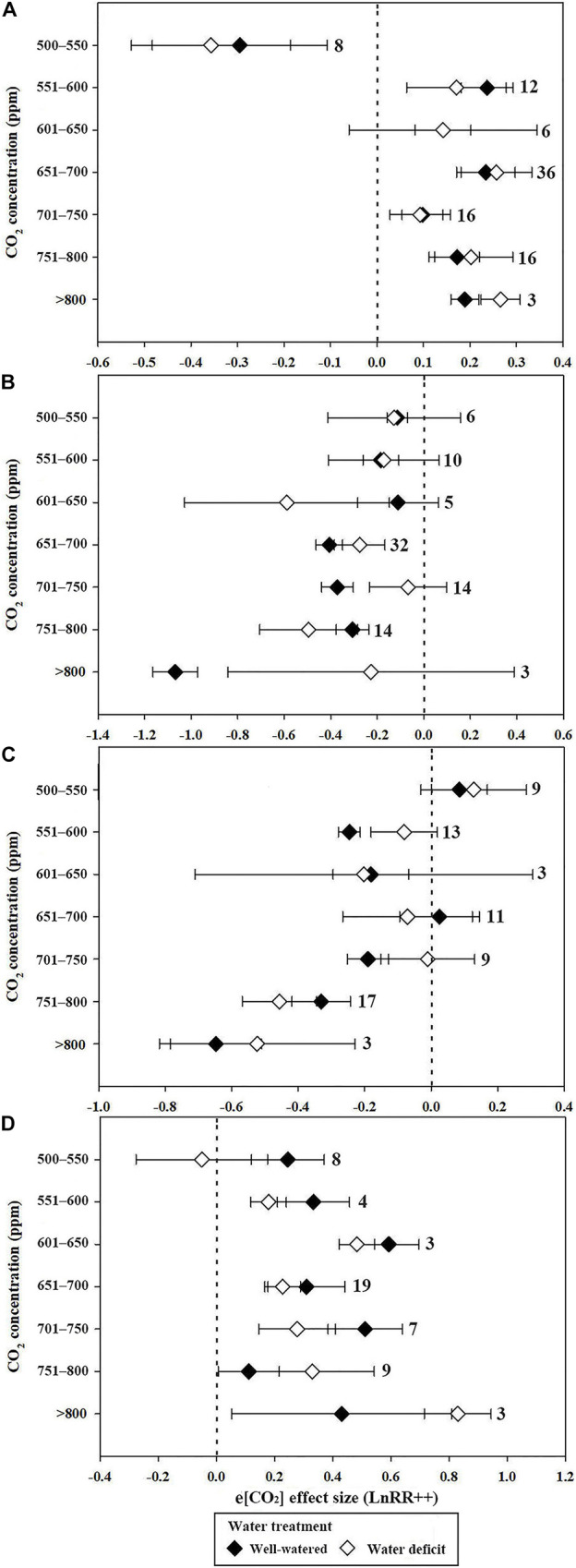
Mean effect sizes of e[CO_2_] on *P*_n_ **(A)**, *G*_*s*_ **(B)**, *T*_r_ **(C)**, and WUE **(D)** under well-watered (black diamond) and water deficit (white diamond) conditions. e[CO_2_] treatments were divided into seven categories as stated on the *y*-axis.

**FIGURE 3:**
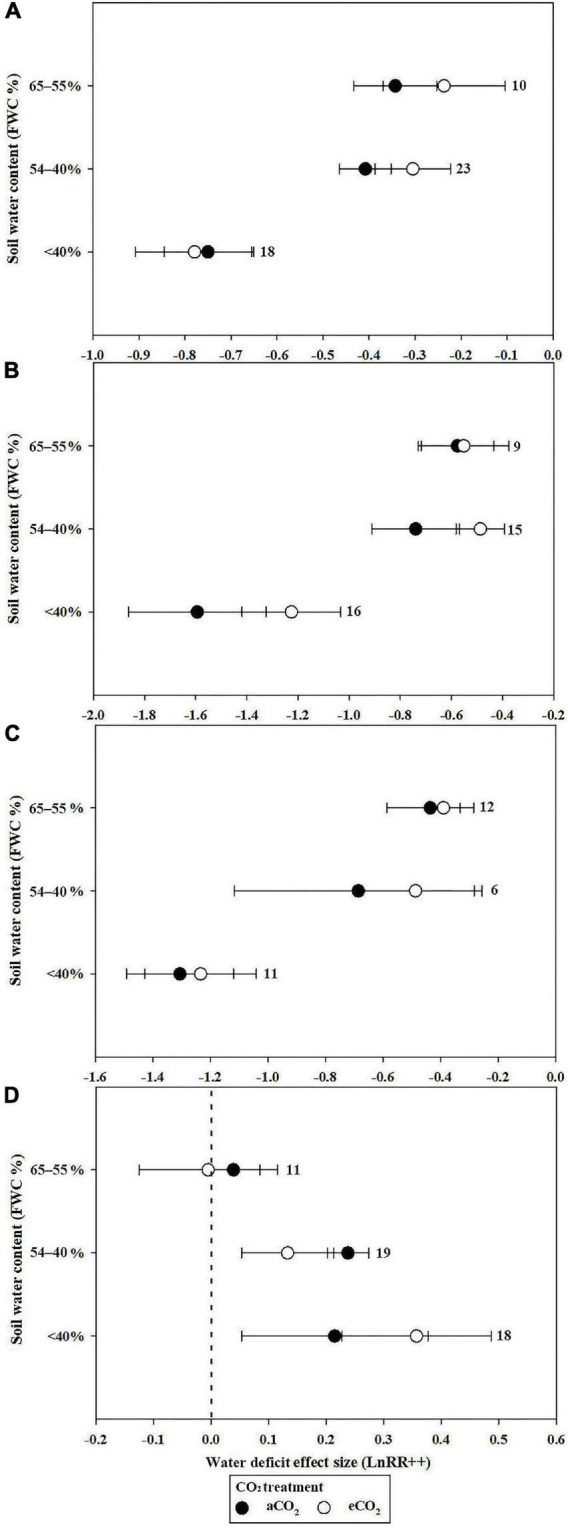
Mean effect sizes of water deficit on *P*_n_ **(A)**, *G*_*s*_ **(B)**, *T*_r_ **(C)**, and WUE **(D)** under a[CO_2_] (black circles) and e[CO_2_] (white circles) treatment. Water-deficit treatments were based on three types of FWC: 65–55, 54–40, and <40%.

The stimulatory effect of increasing [CO_2_] on *P*_n_ reached a maximum at 551 to 600 ppm in well-watered plants. However, the e[CO_2_]-induced increase in *P*_n_ was significantly reduced by water deficit (43.4%) at 551–600 ppm (Figure 2A). Very strong CO_2_ fertilization effects on *P*_n_ were observed when the [CO_2_] increased from 601 to 800 ppm, and became >800 ppm during water deficit compared with the well-watered treatment (Figure 2A). The water deficit effect size magnitudes for *P*_n_ were −0.343, −0.408, and −0.75 at 55–65%, 40–54%, and <40% soil water at a[CO_2_], respectively, indicating that the highest water deficit resulted in the greatest inhibition (Figure 3A).

**FIGURE 4:**
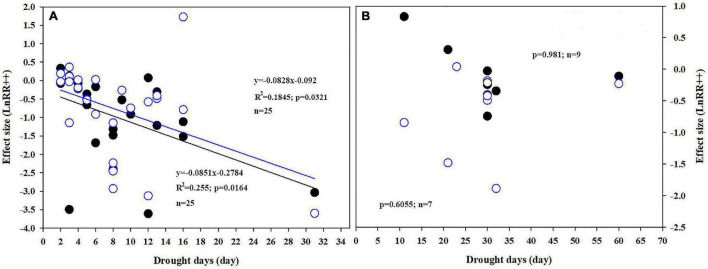
Correlations between drought days and response ratios of *P*_n_ under closed **(A)** and open/semi-open, **(B)** environments under a[CO_2_] (open circles) and e[CO_2_] (closed circles).

Plant *P*_n_ significantly increased by 11.2 and 21.5% when the soil water was between 65–55% and 54–40%, respectively, in conditions of e[CO_2_] compared to a[CO_2_] treatment. However, e[CO_2_] increased the inhibition after water-deficit treatment by 3.9% when the soil water was <40% (Figure 3A). The effect size of *P*_n_ decreased linearly with increasing drought days, with *R*^2^ values of 0.19 (Figure 4A; *p* = 0.0321) and 0.26 (Figure 4A; *p* = 0.0164) at a[CO_2_] and e[CO_2_], respectively, in a closed growing environment. However, there was no significant correlation between plant *P*_n_ response and drought days in open and semi-open environments, possibly owing to the relatively small sample size (*n* = 8) (Figure 4B).

### Stomatal Conductance Responses

Water-deficit treatment reduced the plant *G*_*s*_ for most plants except in shrubs, regardless of the CO_2_ treatment. Tree and crop *G*_*s*_ were significantly reduced by e[CO_2_] after water-deficit treatment by 11.1% (*n* = 53) and 14.5% (*n* = 20), respectively. Additionally, the water-deficit effect sizes of grass and legume *G*_*s*_ were −1.067 and −1.344 under a[CO_2_], which increased by 16.3 and 0.6% compared with that in e[CO_2_] conditions, respectively, (Figure 1). Similarly, e[CO_2_] reduced all plant *G*_*s*_, regardless of the drought treatment, except for shrubs. The e[CO_2_] effect sizes of crop, legume, tree, and grass *G*_*s*_ were −1.229, −1.718, −1.057, and −1.519 under well-watered conditions; and −0.406, −0.376, −0.251, and −0.204 under water-deficit treatments, respectively (Figure 1). Therefore, water deficit reduced the e[CO_2_] response. In contrast, the effect size of shrubs was positive (0.135); however, the 95% CI included zero. The effects of the two water control methods on *G*_*s*_ were also not significant. The interactions of water deficit and e[CO_2_] on *G*_*s*_ were not statistically significant for plant type and control methods of water because the 95% CI included zero.

When the CO_2_ concentration was higher than 650 ppm (651–700, 701–750, 751–800, and >800 ppm), *G*_*s*_ decreases significantly in conditions of well-watered treatment (Figure 2B). Similarly, water-deficit treatment reduced *G*_*s*_. The water-deficit effect size of *G*_*s*_ was −0.577, −0.74, and −1.594 when the FWC was 65–55, 54–40, and <40% after a[CO_2_] treatment, respectively. However, the water-deficit effect size of *G*_*s*_ was −0.552, −0.487, and −1.226 when the FWC was 65–55, 54–40, and <40% after e[CO_2_] treatment (Figure 3B).

### Transpiration Rate Responses

Water deficit reduced the *T*_r_ of C_3_ plants to a greater degree than C_4_ plants, whereas e[CO_2_] reduced the *T*_r_ of C_4_ plants more than that of C_3_ plants. Meanwhile, water deficit reduced the *T*_r_ of grasses, trees, and legumes more than crops regardless CO_2_ concentration. Additionally, water deficit reduced the *T*_r_ by 29.7, 26, 0.52, and 56.5% in OTC, greenhouse, FACE, and growth chambers, respectively, under a[CO_2_] (Figure 1B). However, water deficit increased the *T*_r_ by 9.6% in FACE under e[CO_2_] treatment (Figure 1A). Moreover, e[CO_2_] reduced *T*_r_ regardless of the water condition and CO_2_ fumigation method except for OTC. However, the interactions of drought and e[CO_2_] on *T*_r_ were statistically insignificant for legumes and crop species.

The decrease in *T*_r_ for plants resulted in a much larger decrease in *T*_r_ at >750 ppm CO_2_ compared with 551–750 ppm CO_2_ (Figure 2C). However, there was no significant influence on *T*_r_ at 500–600 ppm CO_2_ (Figure 2C). The water deficit effect size of *T*_r_ was −0.436, −0.68, and −1.306 when FWC was 65–55, 54–40, and <40%, respectively (Figure 3C).

### Leaf-Level Water Use Efficiency Responses

**FIGURE 5:**
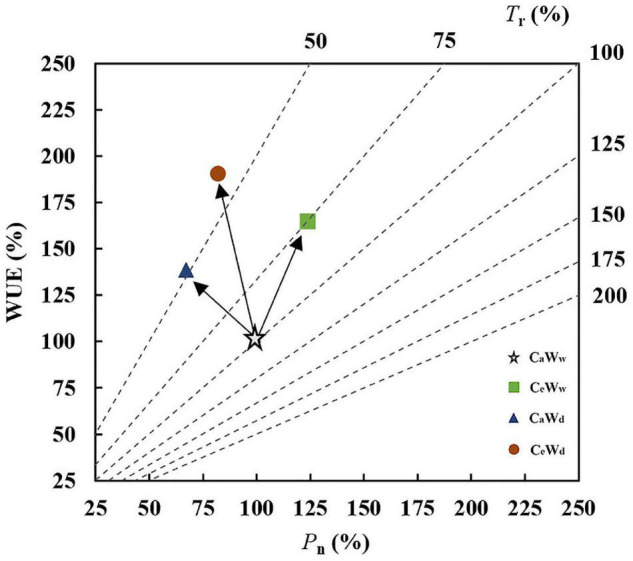
Graphical vector analysis (GVA) showing the effects of e[CO_2_] and water status on *P*_n_, *T*_r_, and WUE (C_*e*_W_*d*_, orange circle; C_*e*_W_*w*_, green box; C_*a*_W_*d*_, blue triangle; and C_*a*_W_*w*_, white star).

**FIGURE 6:**
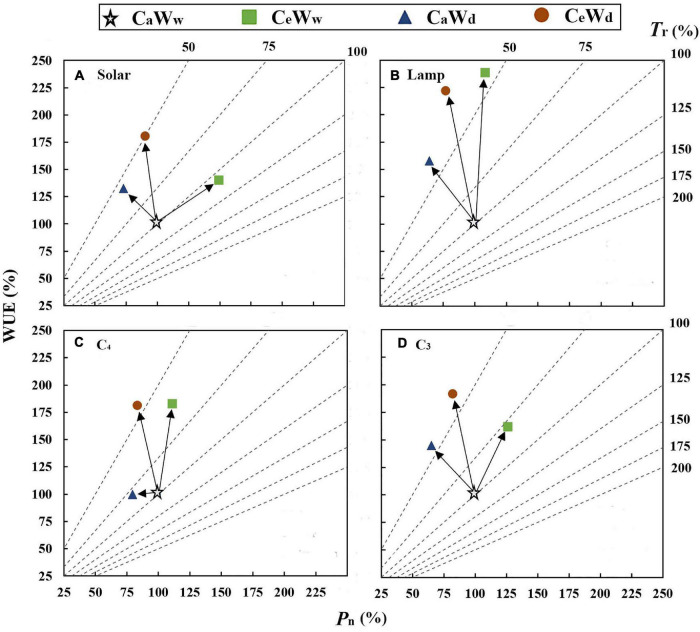
Graphical vector analysis showing the effects of e[CO_2_] and water deficit on *P*_n_, *T*_r_, and WUE (C_*e*_W_*d*_, orange circle; C_*e*_W_*w*_, green box; C_*a*_W_*d*_, blue triangle; and C_*a*_W_*w*_, white star). **(A)** Plants grown under solar radiation; **(B)** plants grown under lamps; **(C)** C_4_ plants; and **(D)** C_3_ plants.

Graphical vector analysis showed that increased leaf-level WUE under C_*e*_W_*w*_ treatment compared to the control (C_*a*_W_*w*_) may be due to the common effect of *P*_n_ and *T*_r_ (Figure 5). However, increased WUE caused by water deficit (C_*a*_W_*d*_) is attributable to the “*T*_r_” positive effect (Figure 5). Water deficit induced a larger increase in WUE under solar grown plants compared to plants grown under a lamp, regardless of [CO_2_]. e[CO_2_]-induced stimulation of WUE was attributable to a “*P*_n_” positive effect when grown in sunlight (Figures 6A,B). There were no significant interaction effects for C_3_ plants in WUE (Figures 1A–C). Water deficit increased the WUE of C_3_ plants by 32.8 and 21.4% in the a[CO_2_] and e[CO_2_] treatments, respectively, but it had no effect on the WUE of C_4_ plants. Instead, water deficit resulted in a parallel decrease in *P*_n_ and *T*_r_ of C_4_ plants (“steady-state” decrease) under a[CO_2_] (Figures 6C,D). Meanwhile, e[CO_2_] increased the WUE of C_3_ and C_4_ plants by 31.7 and 53.3%, respectively, in the well-watered treatments, and by 20 and 95.8%, respectively, in the water-deficit treatments.

Water use efficiency reached a maximum of 0.593 (81.0%) and 0.483 (62.1%) when the CO_2_ concentration was 601–650 ppm in water deficit and well-watered treatments, respectively. The e[CO_2_]-induced increases in WUE were less prominent in the water deficit treatment than in the well-watered treatment at 500–550, 551–600, 601–650, and 651–700 ppm [CO_2_] (0.246 vs. −0.05; 0.334 vs. 0.18; 0.593 vs. 0.483; 0.31 vs. 0.228; and 0.512 vs. 0.278, respectively) (Figure 2D). However, the e[CO_2_]-induced increase in WUE during water deficit treatment was 1.8 and 2.53 times greater than that of the well-watered treatments at 751–800 ppm CO_2_ and >800 ppm CO_2_, respectively (Figure 2D). The water deficit induced increase in WUE in the e[CO_2_] treatment was 47.1% lower than that in the a[CO_2_] treatment when FWC was 54–40%, whereas the increase in WUE in the e[CO_2_] treatment was 78.8% higher than that in the a[CO_2_] treatment when FWC was <40% (Figure 3D). Additionally, there was no significant change in WUE when the FWC was between 55 and 65% (Figure 3D).

## Discussion

### Soil Water Deficit Constrains the Positive e[CO_2_] Effect on Gas Exchange

**FIGURE 7:**
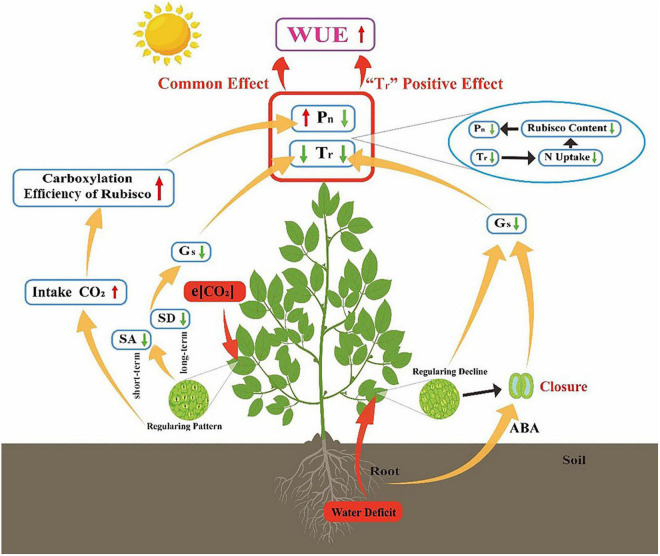
Possible mechanism for soil water deficit constraining the CO_2_ fertilization effect on gas exchange and leaf-level WUE. e[CO_2_] increases carboxylation efficiency of Rubisco by consuming more CO_2_, which boosts *P*_n_. For short-term responses, plants reduce stomatal aperture (SA) while decreasing stomatal density (SD) for long-term response, which both can reduce *G*_*s*_ and *T*_r_. Plant WUE is enhanced owing to the *P*_n_ and *T*_r_ common effect. Water stress induces abscisic acid (ABA) synthesis in the root which leads to stomatal closure and loss of regularity of stomatal distribution. Low *G*_*s*_ and *T*_r_ reduces N acquisition, thus inhibiting *P*_n_. Water deficit-induced increase of WUE is mainly caused by *T*_r_ decrease rather than *P*_n_.

It has been suggested that the positive effects of e[CO_2_] cannot be maintained when other environmental factors (nutrients, temperature, and availability of water) are limited (Carter et al., 2007; Elliott et al., 2014; Fernando et al., 2019). Our meta-analysis results supported our H1 hypothesis that the e[CO_2_]-induced stimulation of *P*_n_ was reduced by water deficit treatment (Figures 1B,C). e[CO_2_] leads to a delay in the onset of drought stress due to increased stomatal closure preventing water loss (Paoletti and Grulke, 2005; Yamaguchi et al., 2019; Jiménez et al., 2020). However, this delay only existed under mild drought stress because further stomatal closure leads to decreases in intercellular CO_2_ and reduces carbon assimilation (Figure 7). e[CO_2_] did not weaken the inhibition by water deficit treatment when soil water was <40% FWC (Figure 3A). This result indicates that the effect of soil water deficit is stronger than that of e[CO_2_] when under severe water-deficit stress. In general, water deficit negatively affected the *P*_n_ of most C_3_ plants due to favoring RuBP oxygenation over carboxylation. Meanwhile, water deficit induced the stomatal closure reduced *T*_r_, thereby inhibiting the uptake of N, which was associated with reductions in the amount and/or activity of Rubisco (Figure 7). Additionally, Zheng et al. (2020) found that the regularity of stomatal distribution pattern was dramatically reduced by e[CO_2_] when winter wheat plants were constrained to moderate and severe stresses, implying that soil moisture conditions partly determined the response of stomatal distribution pattern to e[CO_2_] (Figure 7). The effect size of *P*_n_ decreased linearly with increasing drought days, regardless of [CO_2_], also suggesting that water stress had a greater impact on plant growth than e[CO_2_] when plants are under severe stress.

### Soil Water Deficit Modulated the [CO_2_] Effect on Leaf-Level Water Use Efficiency

Stomatal optimization theory states that stomatal opening to allow CO_2_ uptake inevitably comes at the expense of H_2_O loss (Ferris et al., 2002; Robredo et al., 2010). Therefore, climate change will affect not only the rate of carbon fixation in plants, but also water loss. Evidence was found to support our H2 hypothesis that both water deficit and e[CO_2_] treatment improved leaf-level WUE, but the underlying mechanisms for maintaining high water availability may be different. In general, an increased WUE is a rapid response to drought by closing stomata through root-generated xylem-born ABA (Ferris et al., 2002; Zhang et al., 2018, 2021). Conversely, e[CO_2_] changes stomatal aperture (SA) by increasing intercellular [CO_2_] for a short-term response (Xu et al., 2016; Li S. et al., 2020). Additionally, stomatal density (SD) also decreased when plants were exposed to high [CO_2_] for a long time (Lin et al., 2001; Zhang et al., 2019). Concurrently, an increase on *P*_n_, driven by the improve in increase by improved regularity of stomatal spatial distribution (Xu, 2015; Zheng et al., 2020). This study showed that water deficit and e[CO_2_] significantly increased WUE by 24.5 and 29.3%, respectively (Figure 1A). Increases in WUE under the C_*a*_W_*d*_ treatment are attributable to the “*T*_r_” positive effect (Figure 5), whereas increases in WUE under the C_*e*_W_*w*_ treatment may be due to the common effect of *P*_n_ and *T*_r_ (Figure 7). Meanwhile, water deficit increased the WUE of C_3_ plants by 32.8 and 21.4% in the a[CO_2_] and e[CO_2_] treatments, respectively, implying that the water deficit induced increase of WUE may also be modulated by [CO_2_]. In contrast, e[CO_2_] had a greater effect on WUE of C_4_ plants than C_3_ plants in water deficit treatment, which is consistent with previous studies (Peaarcy and Ehleringer, 1984). C_4_ plants have higher light energy use efficiency because photosynthesis is completed by the combination of vascular bundle sheath cells and mesophyll cells (Peaarcy and Ehleringer, 1984; Ainsworth and Long, 2005). Therefore, C_4_ plants have higher CO_2_ assimilation rates, less transpiration and water loss, and higher WUE compared with C_3_ plants (Peaarcy and Ehleringer, 1984; Conley et al., 2001). In addition, e[CO_2_]-induced stimulation of WUE is attributable to a “*P*_n_” positive effect when growing under sunlight regardless of water concentration. Hence, higher light appears to stimulate the effect of e[CO_2_] on plants more efficiently than lamps.

### Effects of Growing Environment and Plant Type on Plant Gas Exchange Response to e[CO_2_]

It is widely reported that e[CO_2_] stimulates *P*_n_ through the “CO_2_ fertilization effect” because the current CO_2_ concentration limits the photosynthetic ability of plants (Xu, 2015; Fitzgerald et al., 2016). Our meta-analysis demonstrated that the % increase in *P*_n_ by e[CO_2_] treatments depended on the fumigation method; *P*_n_ was stimulated to a greater magnitude when plants were exposed to e[CO_2_] in an open environment than in a closed environment, especially when compared to the growth chamber. In contrast, another meta-analysis studying the effects of e[CO_2_] indicated that the more closely the fumigation conditions mimicked field conditions, the smaller the stimulation of rice yield (Ainsworth, 2008). It is important to note that earlier studies were conducted at lower control [CO_2_] than recent studies owing to changes in atmospheric CO_2_. For example, earlier FACE and chamber studies were conducted at 339 and 330–360 ppm control CO_2_, respectively (Baker et al., 1990; Teramura et al., 1990), whereas recent control experiments used approximately 400 ppm (Zinta et al., 2014; van der Kooi et al., 2016; Zheng et al., 2020). In addition, there were differences in the light conditions used. For example, FACE and OTC rely on natural light, and some greenhouse/glasshouse studies also used natural light but were covered by transparent material at the top, whereas almost all plants growing in growth chambers used lamps as a light source. Enclosed growing environments may be limited through the downregulation of photosynthesis (Ainsworth and Long, 2005; Zhang et al., 2021). Under high light conditions, plants are generally better able to take advantage of increased [CO_2_] due to altered N partitioning within the photosynthetic apparatus to favor light-harvesting complexes (Yu et al., 2012; Kizildeniz et al., 2021). However, at low light levels, light-dependent reactions limit the rate of photosynthesis (Assmann, 1999; Yu et al., 2012). Our meta-analysis supported these conclusions since the *P*_n_ of plants grown under natural light was higher than that under lamps. Therefore, light levels and CO_2_ concentration may contribute to a smaller stimulation of *P*_n_ by e[CO_2_] in a closed–CO_2_ fumigation system.

Our results showed that e[CO_2_] reduced *G*_*s*_, but the extent of the decrease varied according to plant type. The *G*_*s*_ of legumes are more sensitive to [CO_2_] which may be due to the decrease in total K content of legumes in high CO_2_ environments, and the decrease in K^+^ concentration increases stomatal resistance resulting in stomatal closure (Peaarcy and Ehleringer, 1984). Moreover, *G*_*s*_ reached a minimum when the [CO_2_] was >800 ppm, while the *P*_n_ did not decrease, implying that *G*_*s*_ was not the cause of *P*_n_ variation (Figure 1B). However, this result contradicts previous conclusions stating that changing photosynthesis and *G*_*s*_ is evidence of stomatal control over photosynthesis (Lawson and Blatt, 2014; Xu et al., 2016). It is possible that the decreased *G*_*s*_ may be the result rather than the cause of decreased photosynthesis. This may be related to e[CO_2_], resulting in an increase in the concentration of ions and organic molecules; thus, guard cells swell to balance the water potential inside and outside the cell, reducing stomatal openness. Nevertheless, it should be noted that this study did not compare the long-term and short-term fumigation time of plants with high CO_2_ concentration mainly due to the limited data and small sample size in previous literatures. Therefore, further studies with long-term multi-factor experiments are needed to fully understand the mechanisms and processes governing the interactions between e[CO_2_] and water deficit on many plant types for comparing the “long-term” and “short-term” effects of e[CO_2_] on plants under future climate change.

## Conclusion

Our meta-analysis demonstrated that e[CO_2_] generally augmented *P*_n_, but the magnitude of the increase varied depending on the CO_2_ fumigation method and light conditions. The greatest increases occurred when plants were exposed to e[CO_2_] in an open environment under natural light. Previous projections based on the results of earlier e[CO_2_] experiments may underestimate the “CO_2_ fertilization effect” in future global terrestrial ecosystems, because the CO_2_ fertilization effect on plant *P*_n_ may be limited by the enclosed experimental methods and low light. Our results also indicated that both water deficit and e[CO_2_] improved leaf-level WUE. However, e[CO_2_]-induced stimulation of WUE is attributable to the *P*_n_ and *T*_r_ common effect, whereas water deficit induced increases of WUE are attributable to the “*T*_r_” positive effect. Additionally, water deficit may result in a greater impact on the *P*_n_ and WUE than e[CO_2_], that is, the “CO_2_ fertilization effect” may be modulated by soil water conditions under future climate change. Therefore, fumigation conditions that more closely mimic field conditions and multi-factorial experiments such as water availability, high temperature, low N/P, and elevated O_3_ are needed to predict the response of plants to future climate change.

## Data Availability Statement

The datasets presented in this study can be found in online repositories. The names of the repository/repositories and accession number(s) can be found in the article/Supplementary Material.

## Author Contributions

FL, XG, and XZ designed the study. FL and DG performed the literature collection and data extraction. FL performed data analysis and wrote the initial manuscript with additional input from XG and XZ. All authors edited and approved this version of the manuscript.

## Conflict of Interest

The authors declare that the research was conducted in the absence of any commercial or financial relationships that could be construed as a potential conflict of interest.

## Publisher’s Note

All claims expressed in this article are solely those of the authors and do not necessarily represent those of their affiliated organizations, or those of the publisher, the editors and the reviewers. Any product that may be evaluated in this article, or claim that may be made by its manufacturer, is not guaranteed or endorsed by the publisher.

## Footnotes

1
http://apps.webofknowledge.com

2
http://www.cnki.net
