# Supplementary material for: Water Deficit Modulates the CO2 Fertilization Effect on Plant Gas Exchange and Leaf-Level Water Use Efficiency: A Meta-Analysis
Source: Front Plant Sci. 2021 Nov 29;12:775477. doi: 10.3389/fpls.2021.775477 (PMC8667667; doi:10.3389/fpls.2021.775477)
Supplement: Supplementary file 1 [file Data_Sheet_1.docx]

Supplementary Material

Water deficit modulates the CO_2_ fertilization eﬀect on plant gas exchange and leaf-level water use efficiency: A meta-analysis

Fei Li, Dagang Guo, Xiaodong Gao, Xining Zhao

Corresponding author: X. Zhao([zxn@nwafu.edu.cn](mailto:zxn@nwafu.edu.cn))

Table of contents:

Table S1. The overall effect sizes of plants gas exchange which water deficit treatment effect on elevated CO_2_ response

Table S2. Between-group heterogeneity for the effect of explanatory variables in affecting water deficit treatment effect on plant responses to e[CO_2_]

Ref S1. Reference list of studies included in this analysis

| Variable | CO_2_ effect under water deficit treatment | | | | CO_2_ effect under well-watered treatment | | | | Interaction between water deficit and e[CO_2_] | | | |
| --- | --- | --- | --- | --- | --- | --- | --- | --- | --- | --- | --- | --- |
|  | *Q*_t_ | *p*-value | Effect size (%) | CI (%) | *Q*_t_ | *p-*value | Effect size (%) | CI (%) | *Q*_t_ | *p*-value | Effect size (%) | CI (%) |
| *P*_n_ | 10082.804 | <0.001 | 16.4 | 0.059 to 0.245 | 189.598 | <0.001 | 11.9 | -0.015 to 0.171 | 9965.311 | <0.001 | 8.3 | 0.039 to 0.254 |
| *G*_s_ | 385.476 | <0.001 | 23.4 | -0.394 to 0.127 | 4697.764 | <0.001 | 28.5 | -0.419 to 0.251 | 1729814.295 | <0.001 | 3.8 | -0.128 to 0.196 |
| *T*_r_ | 5999.664 | <0.001 | 14.4 | -0.297 to 0.065 | 2874.528 | <0.001 | 19.1 | -0.292 to 0.132 | 64281.812 | <0.001 | 4.1 | -0.004 to 0.168 |
| WUE | 170.061 | <0.001 | 28.8 | 0.033 to 0.363 | 7966.307 | <0.001 | 29.3 | 0.117 to 0.43 | 1544.405 | <0.001 | 7.6 | -0.244 to 0.107 |

**Table S1.** Water deficit treatment effect on plant responses to e[CO_2_]. Results were based on log-transformed response ratios. Effect sizes were reported as the antilog r converted to the mean percentage change from a baseline treatment [(r−1) × 100]. Qt is a statistic based on a chi-squared test used to examine the heterogeneity across case studies. A significant Qt value indicated that effect sizes are not equal across studies, implying that other explanatory variables influence the results. CI refers to the 95% confidence interval of the effect size. Response variables are: net photosynthetic rate (*P*_n_), stomatal conductance (*G*_s_), transpiration rate (*T*_r_), and leaf-level water use efficiency (WUE).

| Variable | CO_2_ effect under water deficit treatment | | | CO_2_ effect under well-watered treatment | | | Water deficit under a[CO_2_] | | | Water deficit under e[CO_2_] | | Interaction between water deficit and e[CO_2_] | | | |
| --- | --- | --- | --- | --- | --- | --- | --- | --- | --- | --- | --- | --- | --- | --- | --- |
|  | *Q*_m_ | *p*-value | *Q*_m_ | | *p*-value | *Q*_m_ | | *p*-value | *Q*_m_ | | *p*-value | *Q*_m_ | | *p*-value | |
| Photosynthetic pathway | | | | | | | | | | | | | | | |
| *P*_n_ | 1.334 | 0.248 | 0.232 | | 0.63 | 0.871 | | 0.351 | 0.313 | | 0.576 | 1.334 | 0.248 | |  |
| *G*_s_ | 0.001 | 0.977 | 1.945 | | 0.163 | 0.27 | | 0.603 | 3.533 | | 0.062 | 0.27 | 0.603 | |  |
| *T*_r_ | 0.121 | 0.728 | 5.246 | | 0.022* | 6.208 | | 0.013* | 10.496 | | 0.001* | 0.585 | 0.445 | |  |
| WUE | 5.648 | 0.017* | 0.527 | | 0.468 | 6351 | | 0.011* | 1.018 | | 0.313 | 0.385 | 0.535 | |  |
| Plant type | | | | | | | | | | | | | | | |
| *P*_n_ | 2.796 | 0.592 | 6.917 | | 0.14 | 0.286 | | 0.951 | 0.74 | | 0.946 | 2.47 | | 0.65 | |
| *G*_s_ | 1.592 | 0.81 | 3.066 | | 0.547 | 1.194 | | 0.879 | 11.659 | | 0.002* | 4.957 | | 0.292 | |
| *T*_r_ | 0.767 | 0.857 | 2.145 | | 0.342 | 12.651 | | 0.005* | 6.111 | | 0.106 | 2.477 | | 0.479 | |
| WUE | 1.423 | 0.84 | 5.861 | | 0.21 | 3.888 | | 0.421 | 4.494 | | 0.344 | 0.795 | | 0.593 | |
| Control methods of water | | | | | | | | | | | | | | | |
| *P*_n_ | 0.386 | 0.534 | 5.804 | | 0.016* | 0.266 | | 0.001* | 0.168 | | 0.682 | 0.782 | | 0.377 | |
| *G*_s_ | 0.368 | 0.544 | 0.023 | | 0.88 | 1.022 | | 0.312 | 0.923 | | 0.337 | 0.477 | | 0.49 | |
| *T*_r_ | 0.000 | 0.099 | 0.032 | | 0.859 | 0.286 | | 0.591 | 0.002 | | 0.961 | 1.816 | | 0.178 | |
| WUE | 0.689 | 0.406 | 1.212 | | 0.271 | 0.569 | | 0.45 | 0.028 | | 0.866 | 2.57 | | 0.109 | |
| Methods of fumigation | | | | | | | | | | | | | | | |
| *P*_n_ | 1.72 | 0.787 | 15.9718 | | 0.003* | 0.176 | | 0.176 | 5.011 | | 0.71 | 6.578 | | 0.16 | |
| *G*_s_ | 8.066 | 0.089 | 6.652 | | 0.155 | 8.34 | | 0.08 | 6.965 | | 0.138 | 9.021 | | 0.061 | |
| *T*_r_ | 1.171 | 0.76 | 5.966 | | 0.113 | 14.158 | | 0.003* | 9.986 | | 0.019* | 3.242 | | 0.356 | |
| WUE | 2.403 | 0.662 | 3.117 | | 0.538 | 3.481 | | 0.481 | 0.96 | | 0.811 | 2.105 | | 0.716 | |
| Light source | | | | | | | | | | | | | | | |
| *P*_n_ | 0.097 | 0.756 | 14.388 | | 0.001* | 0.214 | | 0.644 | 2.289 | | 0.13 | 6.745 | | 0.009* | |
| *G*_s_ | 4.071 | 0.044* | 2.733 | | 0.098 | 0.167 | | 0.683 | 3.762 | | 0.052 | 6.717 | | 0.01* | |
| *T*_r_ | 1.128 | 0.288 | 3.17 | | 0.075 | 9.616 | | 0.002* | 4.317 | | 0.038* | 0.769 | | 0.381 | |
| WUE | 0.228 | 0.623 | 2.359 | | 0.122 | 6.288 | | 0.012* | 0.237 | | 0.626 | 1.304 | | 0.254 | |

**Table S2.** Between-group heterogeneity of explanatory variables affecting water deficit treatment on plant responses to e[CO_2_]. Explanatory variables include photosynthetic pathways, plant type, control methods of water, methods of fumigation and light source. *Q*_m_ refers to the test of explanatory variables effect, with its *p*-value < 0.05 suggesting significant differences between groups (*). CI refers to the 95% confidence interval of the effect size.

**Ref S1.** Reference list of studies included in this analysis

1. Robredo, A., Perez-Lopez, U., de la Maza, H. S., Gonzalez-Moro, B., Lacuesta, M., Mena-Petite, A., et al. (2007). Elevated CO_2_ alleviates the impact of drought on barley improving water status by lowering stomatal conductance and delaying its effects on photosynthesis. *Environmental and Experimental Botany, 59*(3), 252–263. doi:10.1016/j.envexpbot.2006.01.001

2. Parvin, S., Uddin, S., Tausz-Posch, S., Fitzgerald, G., Armstrong, A., Tausz, M. (2019). Elevated CO_2_ improves yield and N_2_ fixation but not grain N concentration of fababean (*Vicia faba* L.) subjected to terminal drought. *Environmental and Experimental Botany,* *165*, 161–173. [doi:10.1016/j.envexpbot.2019.06.003](https://doi.org/10.1016/j.envexpbot.2019.06.003)

3. Vu, J. C. V., Allen, L. H. (2009). Growth at elevated CO_2_ delays the adverse effects of drought stress on leaf photosynthesis of the C_4_ sugarcane. *Journal of Plant Physiology, 166*(2), 107–116. Doi: 10.1016/j.jplph.2008.02.009

4. Aranda, I., Cadahia, E., de Simon, B. F. (2020). Leaf ecophysiological and metabolic response in Quercus pyrenaica Willd seedlings to moderate drought under enriched CO_2_ atmosphere. *Journal of Plant Physiology,* 244. doi: 10.1016/j.jplph.2019.153083

5. Schutz, M., Fangmeier, A. (2001). Growth and yield responses of spring wheat (*Triticum aestivum* L. cv. Minaret) to elevated CO_2_ and water limitation. *Environmental Pollution, 114*(2), 187–194. doi:10.1016/s0269-7491(00)00215-3

6. Li, D., Liu, H., Qiao, Y., Wang, Y., Cai, Z., Dong, B., et al. (2013). Effects of elevated CO_2_ on the growth, seed yield, and water use efficiency of soybean (*Glycine max* (L.) Merr.) under drought stress. *Agricultural Water Management, 129*, 105–112. doi: 10.1016/j.agwat.2013.07.014

7. Avila, R.T., Amanda.Cardosoa, A. A., Almeida, W. L., Costaa, L. C., Machadoa, K. L. G., Marcela L.Barbosa, M.L.,et al. (2020). Coffee plants respond to drought and elevated CO_2_ through changes in stomatal function, plant hydraulic conductance, and aquaporin expression. *Environmental and Experimental Botany,* 177. doi: 10.1016/j.envexpbot.2020.104148

8. Li, B., Feng, Y., Zong, Y., Zhang, D., Hao, X., Li, P. (2020). Elevated CO_2_-induced changes in photosynthesis, antioxidant enzymes and signal transduction enzyme of soybean under drought stress. *Plant Physiology and Biochemistry, 154*, 105–114. doi: 10.1016/j.plaphy.2020.05.039

10. Harnos, N., Bencze, S., Janda, T., Juhasz, A., Veisz, O. (2002). Interactions between elevated CO_2_ and water stress in two winter wheat cultivars differing in drought resistance. *Cereal, Research Communication*

11. Chen, Y., Yu, J., Huang, B. (2015). Effects of elevated CO_2_ concentration on water relations and photosynthetic responses to drought stress and recovery during rewatering in Tall Fescue. *Journal of the American Society for Horticultural Science, 140*(1), 19–26. doi: 0.21273/jashs.140.1.19

12. Souza, J. P., Melo, N. M. J., Pereira, E. G., Halfeld, A. D., Gomes, I. N., Prado, C. H. B. A. (2016). Responses of woody Cerrado species to rising atmospheric CO_2_ concentration and water stress: gains and losses. *Functional Plant Biology, 43*(12), 1183–1193. doi: 10.1071/fp16138

13. Zheng, Y., He, C., Guo L., Hao, L., Cheng, D., Li F., et al. (2020). Soil water status triggers CO_2_ fertilization effect on the growth of winter wheat (*Triticum aestivum)*. *Agricultural and Forest Meteorology, 291*. Doi: 10.1016/j.agrformet.2020.108097

14. Sgherri, C. L. M., Quartacci, M. F., Menconi, M., Raschi, A., & Navari-Izzo, F. (1998). Interactions between drought and elevated CO_2_ on alfalfa plants. *Journal of Plant Physiology, 152*(1), 118–124. Doi: 10.1016/s0176-1617(98)80110-7

15. Zhao, N., Meng, P., He, Y., Yu, X. (2017). Interaction of CO_2_ concentrations and water stress in semiarid plants causes diverging response in instantaneous water use efficiency and carbon isotope composition. *Biogeosciences, 14*(14), 3431–3444. doi:10.5194/bg-14-3431-2017

16. Zhang, Y., Yu, X., Chen, L., Jia, G. (2019). Whole-plant instantaneous and short-term water-use efficiency in response to soil water content and CO_2_ concentration. *Plant and Soil, 444*(1–2), 281-298. doi: 10.1007/s11104-019-04277-6

17. Wijewardana, C., Henry, W. B., Gao, W., Reddy, K. R. (2016). Interactive effects on CO_2_, drought, and ultraviolet-B radiation on maize growth and development. *Journal of Photochemistry and Photobiology B-Biology, 160*, 198–209. doi: 10.1016/j.jphotobiol.2016.04.004

18. Wang, H., Zhou, G. S., Jiang, Y. L., Shi, Y. H., Xu, Z. Z. (2017). Photosynthetic acclimation and leaf traits of *Stipa bungeana* in response to elevated CO_2_ under five different watering conditions. *Photosynthetica, 55*(1), 164–175. doi:10.1007/s11099-016-0239-1

19. Ambebe, T. F., Dang, Q.-L. (2009). Low moisture availability inhibits the enhancing effect of increased soil temperature on net photosynthesis of white birch (*Betula papyrifera*) seedlings grown under ambient and elevated carbon dioxide concentrations. *Tree Physiology, 29*(11), 1341–1348. doi:10.1093/treephys/tpp079

20. Tom-Dery, D., Eller, F., Fromm, J., Jensen, K. Reisdorff, C. (2019). Elevated CO_2_ does not offset effects of competition and drought on growth of shea (*Vitellaria paradoxa* C.F. Gaertn.) seedlings. *Agroforest System, 93*(5), 1807–1819. doi:10.1007/s10457-018-0286-7

21. Zhou, R., Yu, X., Kjaer, K. H., Rosenqvist, E., Ottosen, C.-O., Wu, Z. (2015). Screening and validation of tomato genotypes under heat stress using F-v/F-m to reveal the physiological mechanism of heat tolerance. *Environmental and Experimental Botany, 118*, 1–11. doi: 10.1016/j.envexpbot.2015.05.006

22. Je, S.-M., Woo, S. Y., Lee, S. H., Kwak, M. J., Lee, T. Y., Kim, S. H. (2018). Combined effect of elevated CO_2_ concentration and drought on the photosynthetic apparatus and leaf morphology traits in seedlings of yellow poplar. *Ecological Research, 33*(2), 403–412. doi:10.1007/s11284-017-1495-7

23. Oliveira, M. F., Marenco, R. A. (2019). Photosynthesis and biomass accumulation in *Carapa surinamensis* (Meliaceae) in response to water stress at ambient and elevated CO_2_. *Photosynthetica, 57*(1), 137–146. doi:10.32615/ps.2019.023

24. Liu, J., Kang, S., Davies, W. J., Ding, R. (2020). Elevated CO_2_ alleviates the impacts of water deficit on xylem anatomy and hydraulic properties of maize stems. *Plant*, *Cell and Environment, 43*(3), 563–578. doi:10.1111/pce.13677

25. Faralli, M., Grove, I.G., Hare, M.C., Kettlewell, P.S. Fiorani, F. (2017). Rising CO_2_ from historical concentrations enhances the physiological performance of *Brassica napus* seedlings under optimal water supply but not under reduced water availability. *Plant, Cell and Environment, 40*(2): 317–325. doi: 10.1111/pce.12868

26. Duan, H., Duursma, R.A., Huang, G., Smith RA., Tissue DT. (2014). Elevated [CO_2_] does not ameliorate the negative effects of elevated temperature on drought-induced mortality in *Eucalyptus radiata* seedlings. *Plant, Cell and Environment, 37*(7): 1598–1613. doi: 10.1111/pce.12260

27. Liu, X., Zhang, H., Wang, J., Wu, X., Ma, S., Xu, Z., et al. (2019). Increased CO_2_ concentrations increasing water use efficiency and improvement PSII function of mulberry seedling leaves under drought stress. *Journal of Plant Interactions, 14*(1), 213–223. doi: 10.1080/17429145.2019.1603405

28. Lewis, J. D., Smith, R. A., Ghannoum, O., Logan, B. A., Phillips, N. G., Tissue, D. T. (2013). Industrial-age changes in atmospheric CO_2_ and temperature differentially alter responses of faster- and slower-growing Eucalyptus seedlings to short-term drought. *Tree Physiology, 33*(5), 475–488. doi: 10.1093/treephys/tpt032

29. Lee, S. H., Woo, S. Y., Je, S. M. (2015). Effects of elevated CO_2_ and water stress on physiological responses of *Perilla frutescens* var. *japonica* HARA. *Plant Growth Regulation, 75*(2), 427–434. doi: 10.1007/s10725-014-0003-0

30. Lahive, F., Hadley, P., Daymond, A. J. (2018). The impact of elevated CO_2_ and water deficit stress on growth and photosynthesis of juvenile cacao (*Theobroma cacao* L.). *Photosynthetica, 56*(3), 911–920. doi: 10.1007/s11099-017-0743-y

31. Asif, M., Yilmaz, O., Ozturk, L. (2017). Potassium deficiency impedes elevated carbon dioxide-induced biomass enhancement in well-watered or drought-stressed bread wheat. *Journal of Plant Nutrition and Soil Science, 180*(4), 474–481. https://doi.org/10.1002/jpln.201600616

32. Schmid, I., Franzaring, J., Mueller, M., Brohon, N., Calvo, O. C., Hoegy, P., Fangmeier, A. (2016). Effects of CO_2_ Enrichment and Drought on Photosynthesis, Growth and Yield of an Old and a Modern Barley Cultivar. *Journal of Agronomy and Crop Science, 202*(2), 81–95. doi:10.1111/jac.12127

33. Centritto, M., Lee, H.S.J. Jarvis, P.G. (1999). Interactive effects of elevated CO_2_ and drought on cherry (*Prunus avium*) seedlings - I. Growth, whole-plant water use efficiency and water loss. *New Phytologist, 141*(1)*,* 129–140. doi: 10.1046/j.1469-8137.1999.00326.x

34. Wertin, T. M., McGuire, M. A., Teskey, R. O. (2010). The influence of elevated temperature, elevated atmospheric CO_2_ concentration and water stress on net photosynthesis of loblolly pine (*Pinus taeda* L.) at northern, central and southern sites in its native range. *Global Change Biology, 16*(7), 2089–2103. doi: 10.1111/j.1365-2486.2009.02053.x

35. Serraj, R., Allen, L. H., Sinclair, T. R. (1999). Soybean leaf growth and gas exchange response to drought under carbon dioxide enrichment. *Global Change Biology, 5*(3), 283–291. doi:10.1046/j.1365-2486.1999.00222.x

36. Hebbar, K. B., Apshara, E., Chandran, K. P., Prasad, P. V. V. (2020). Effect of elevated CO_2_, high temperature, and water deficit on growth, photosynthesis, and whole plant water use efficiency of cocoa (*Theobroma cacao* L.). *International Journal of Biometeorology, 64*(1), 47–57. doi:10.1007/s00484-019-01792-0

37. Hamerlynck, E. P., Huxman, T. E., Loik, M. E., Smith, S. D. (2000). Effects of extreme high temperature, drought and elevated CO_2_ on photosynthesis of the Mojave Desert evergreen shrub, Larrea tridentata. *Plant Ecology, 148*(2), 183–193. doi:10.1023/a:1009896111405

38. Bauweraerts, I., Wertin, T. M., Ameye, M., McGuire, M. A., Teskey, R. O., Steppe, K. (2013). The effect of heat waves, elevated CO_2_ and low soil water availability on northern red oak (*Quercus rubra* L.) seedlings. *Global Change Biology, 19*(2), 517–528. doi:10.1111/gcb.12044

39. Souza, J. P., Jesus Melo, N. M., Halfeld, A. D., Vieira, K. I. C., Rosa, B. L. (2019). Elevated atmospheric CO_2_ concentration improves water use efficiency and growth of a widespread Cerrado tree species even under soil water deficit. *Acta Botanica Brasilica, 33*(3), 425–436. doi:10.1590/0102-33062018abb0272

40. Fravolinil, A., Williams, D. G., Thompson, T. L. (2002). Carbon isotope discrimination and bundle sheath leakiness in three C_4_ subtypes grown under variable nitrogen, water and atmospheric CO_2_ supply. *Journal of Experimental Botany, 53*(378), 2261–2269. doi:10.1093/jxb/erf084

41. Tezara, W., Mitchell, V., Driscoll, S. P., Lawlor, D. W. (2002). Effects of water deficit and its interaction with CO_2_ supply on the biochemistry and physiology of photosynthesis in sunflower. *Journal of Experimental Botany, 53*(375), 1781–1791. doi:10.1093/jxb/erf021

42. Cruz, J. L., Alves, A. A. C., LeCain, D. R., Ellis, D. D., Morgan, J. A. (2016). Elevated CO_2_ concentrations alleviate the inhibitory effect of drought on physiology and growth of cassava plants. *Scientia Horticulturae, 210*, 122–129. doi:10.1016/j.scienta.2016.07.012

43. Broughton, K. J., Smith, R. A., Duursma, R. A., Tan, D. K. Y., Payton, P., Bange, M. P., et al. (2017). Warming alters the positive impact of elevated CO_2_ concentration on cotton growth and physiology during soil water deficit. *Functional Plant Biology, 44*(2), 267–278. doi: 10.1071/fp16189

44. Robredo, A., Perez-Lopez, U., Lacuesta, M., Mena-Petite, A., Munoz-Rueda, A. (2010). Influence of water stress on photosynthetic characteristics in barley plants under ambient and elevated CO_2_ concentrations. *Biologia Plantarum, 54*(2), 285–292. doi: 10.1007/s10535-010-0050-y

45. Arp, W. J., Van Mierlo, J. E. M., Berendse, F., Snijders, W. (1998). Interactions between elevated CO_2_ concentration, nitrogen and water: effects on growth and water use of six perennial plant species. *Plant, Cell and Environment, 21*(1), 1–11. doi: 10.1046/j.1365-3040.1998.00257.x

46. Picon-Cochard, C., Guehl, J. M. (1999). Leaf gas exchange and carbohydrate concentrations in Pinus pinaster plants subjected to elevated CO_2_ and a soil drying cycle. *Annals of Forest Science, 56*(1), 71–76. doi:/10.1051/forest:19990109

47. Roden, J. S., Ball, M. C. (1996). Growth and photosynthesis of two eucalypt species during high temperature stress under ambient and elevated CO_2_. *Global Change Biology, 2*(2), 115–128. doi:10.1111/j.1365-2486.1996.tb00056.x

48. Fan, X., Cao, X., Zhou, H., Hao, L., Dong, W., He, C., et al. (2020). Carbon dioxide fertilization effect on plant growth under soil water stress associates with changes in stomatal traits, leaf photosynthesis, and foliar nitrogen of bell pepper (*Capsicum annuum* L.). *Environmental and Experimental Botany, 179*. doi: 10.1016/j.envexpbot.2020.104203

49. Thruppoyil, S.B. Ksiksi, T. (2020). Time-dependent stomatal conductance and growth responses of Tabernaemontana divaricata to short-term elevated CO_2_ and water stress at higher than optimal growing temperature. *Current Plant Biology, 22*: 100127-Article No.: 100127. doi: 10.1016/j.cpb.2019.100127

50. Li, F. S., Kang, S. Z., Zhang, J. H., Cohen, S. (2003). Effects of atmospheric CO_2_ enrichment, water status and applied nitrogen on water- and nitrogen-use efficiencies of wheat. *Plant and Soil, 254*(2), 279–289. doi: 10.1023/a:1025521701732

51. Lui, J., Johannes, H.C. (2015). Responses of plant photosynthesis, growth and water use efficiency of *Auena sativa* to droughtunder different CO_2_ concentration. *Pratacultural Scienc*e, *32*(7): 1116–1123.

52. Han, Y.X., Li, L.Q., Wang, S.J., Mo, Y., Qin, S.S., Xu, M.M., Zhang, Y.Q. (2020). Effects of increased CO_2_ concentration and regulated deficit irrigation on growth and photosynthetic characteristics of different maize varieties. *Water Saving Irrigation, 12*(83) :83–87.

53. Xiao, L., Liu, G. B., Li, P., Xue, S. (2017). Responses of photosynthesis and non-structural carbohydrates of *Bothriochloa* *ischaemum* to doubled CO_2_ concentration and drought stress. *Journal of Plant Nutrition and Fertilizer, 23*(2): 389-397.

54. Li, B.Y., Wang, N., Hao, X.Y., Li, P. (2019). Effects of interaction between elevated atmospheric CO_2_ concentration and drought on photosynthesis of soybean. *Journal of Shanxi Agricultural Sciences, 2019, 7*(2): 222–225, 258.
